# Anti-N-Methyl-D-Aspartate Receptor Encephalitis Associated with Ovarian Teratoma in South China-Clinical Features, Treatment, Immunopathology, and Surgical Outcomes of 21 Cases

**DOI:** 10.1155/2021/9990382

**Published:** 2021-05-21

**Authors:** Huiyun Jiang, Huixia Ye, Yifeng Wang, Yunhui Li, Ying Wang, Xiaomao Li

**Affiliations:** ^1^Department of Gynecology, Third Affiliated Hospital, Sun Yat-sen University, Guangzhou 510630, China; ^2^Department of Gynecology and Obstetrics, Zhujiang Hospital, Southern Medical University, Guangzhou 510000, China

## Abstract

**Objective:**

To study the clinical characteristics and surgical outcomes of anti-NMDAR encephalitis and the immunopathology of associated teratomas.

**Methods:**

Twenty-one patients were enrolled in this retrospective study, who were diagnosed with anti-NMDAR encephalitis with ovarian teratoma and admitted to two tertiary hospitals in South China from July 2014 to December 2019. The clinical data of patients were reviewed. Comparisons were made between the patients with different outcomes after surgery. Immunohistochemical analyses of associated ovarian teratomas were performed.

**Results:**

The mean age of the patients was 24.33 ± 5.12 years. The peak seasons of disease onset were autumn and winter (30.61% and 32.65%). The symptoms could be divided into 8 categories, including psychiatric abnormalities, seizures, movement dysfunction, consciousness disorders, autonomic dysregulation, speech disturbance, central hypoventilation, and memory deficits. All patients developed four or more categories of symptoms within the first four weeks. Twelve patients (57.1%) had a maximum mRS of 5, and 11 patients (52.4%) were admitted to ICU. Twenty patients received surgery, and only 3 patients were diagnosed pathologically with immature ovarian teratomas, while the other 17 patients had mature ovarian teratomas. After surgery, 17 patients (85.0%) got clinical improvement. The central hypoventilation symptom and mature ovarian teratomas were associated with surgical outcome. Immunohistochemical analysis revealed that there were NMDAR-positive neural tissues in all 8 teratomas and in which 3 cases also contained large numbers of NMDAR-positive sebaceous glands and squamous epithelial tissues.

**Conclusion:**

The disease is of high prevalence in autumn and winter. The central hypoventilation symptom and mature ovarian teratomas were associated with surgical outcome. NMDAR-positive neural tissue is not the only etiological factor of encephalitis. We speculate that encephalitis development in some patients may result from NMDAR expression in sebaceous glands and squamous epithelial tissues.

## 1. Introduction

Anti-N-methyl-D-aspartate receptor (NMDAR) encephalitis belongs to immune-mediated disorder with prominent neuropsychiatric symptoms, which is serious and potentially lethal. It was firstly identified by Dalmau et al. [1]. Along with the progress of anti-NMDAR antibody detection and the growing attention paid to the disease, related researches began to rise rapidly. Several autoimmune diseases are known to have well-established seasonal variation. However, there is still little knowledge about the anti-NDMAR encephalitis's onset season.

Teratoma is a confirmed trigger of anti-NMDAR encephalitis [2]. About 50% of the female patients with anti-NMDAR encephalitis were identified to have teratoma [3]. And Dalmau et al. [1] founded neural tissue with strong expression of NR2 subunits in the patients' teratoma, which could react with patients' antibodies. Thus, autoimmune factor is widely considered to be an important etiology of anti-NMDAR encephalitis. However, some patients' teratomas lack neural tissue, and the immunopathology of the associated teratomas needs to be further studied.

The therapeutic methods of anti-NMDAR encephalitis involve immunotherapy and tumor section. First-line immunotherapy is the first choice, including corticosteroids, intravenous immunoglobulins, and plasma exchange. Second-line immunotherapy includes rituximab, cyclophosphamide, and mycophenolate mofetil [4]. Tumor section is usually recommended for patients with ovarian tumors, as studies reported that tumor section was associated with a lower rate of relapse in patients with teratoma compared to patients without teratoma [5]. But it is still lack of study focusing on the influence factors of surgical outcome.

This study is aimed at characterizing the onset season, clinical presentations, auxiliary examinations, treatment experience, immunopathology of teratomas, and prognosis of anti-NMDAR encephalitis with ovarian teratoma and comparing the difference in clinical features of patients with or without clinical improvement after surgery.

## 2. Materials and Methods

### 2.1. Patients

Patients diagnosed with anti-NDAMR encephalitis with ovarian teratoma, who were admitted into the Third Affiliated Hospital, Sun Yat-sen University, and Zhujiang Hospital, Southern Medical University, Guangzhou, China, from July 1, 2014 to December 30, 2019, were enrolled in this study, which was approved by the ethics committee of the Third Affiliated Hospital, Sun Yat-sen University (No. [2017]2-198). Written consent was provided by the patients or their representatives.

The inclusion criteria were as follows: (1) at least one of the symptoms, including psychiatric abnormalities, seizures, speech disturbance, dyskinesias, disturbance of consciousness, autonomic dysfunction, or central hypoventilation; (2) positive anti-NMDAR antibody in cerebrospinal fluid (CSF); (3) imaging findings of ovarian teratoma. All patients were diagnosed with anti-NMDAR encephalitis according to the criteria suggested by Dalmau et al. [2]. The fixed cell-based indirect immunofluorescence test (OUMENG FA 112d-51) was used to detect the anti-NMDAR antibodies in CSF or serum samples.

### 2.2. Data Collection

We collected demographic details, clinical features, auxiliary examinations and imaging examinations, treatments, and prognosis of patients included. Clinical features were divided into eight categories: psychiatric abnormalities, seizures, memory deficits, movement dysfunction, autonomic dysregulation, speech disorder, central hypoventilation, and loss of consciousness.

### 2.3. Outcome Evaluation

The modified Rankin Scale (mRS) was used to evaluate the severity of illness and outcome of patients [4, 6]. An mRS score of 0-2 was considered as good outcome.

A decrease in mRS ≥ 1 was defined as clinical improvement. No change in mRS or mRS ≥ 4 for 1 month after surgery was defined as having no clinical improvement. The new onset or worsening of symptoms occurring after two months of stabilization was considered as disease relapse [5]. Patients received prognosis evaluation at defined time points (1 week, 1 month, 3 months, 6 months, and 12 months after surgery).

### 2.4. Immunohistochemistry

Immunohistochemistry was performed on formalin-fixed, paraffin-embedded specimens. Slides of paraffin-embedded tumors were deparaffinized in xylene for 5 min and rehydrated in ethanol (4 min for 100%, 4 min for 95%, 4 min for 85%, and 4 min for 70%) and then washed by PBS for 2 min. Sections were pretreated with EDTA buffer pH 8.0 to retrieve antigens and washed three times with PBS. Sections were serially incubated with 3% H_2_O_2_ for 10 min and blocked with goat serum (Boster, AR0009) for 30 min. Sections were incubated at 4°C overnight with primary antibodies as follows: anti-NDMAR1 antibody (1 : 200; Abcam ab52177), anti-NMDAR2A (1 : 25; Abcam ab118587), anti-NMDAR2B (1 : 25; Novus NB100-74475), and anti-MAP2(1 : 250; Abcam, ab183093). Then, sections were washed with PBS for three times and incubated with rabbit or mouse normal serum for 30 min and labeled polymer-HRP anti-rabbit or anti-mouse. DAB was used to stain the sections.

### 2.5. Statistical Analysis

SPSS version 20.0 was used to perform Statistical analyses. *p* values <0.05 (two-sided) were considered significant. Baseline parameters were presented using descriptive statistics. Continuous variables were analyzed with an independent sample *t*-test. Contingency tables were analyzed using Pearson's chi-square test or Fisher's two-sided exact test when appropriate. Factors influencing surgical outcome were assessed by multivariable binary logistic regression.

## 3. Results

**Figure 1:**
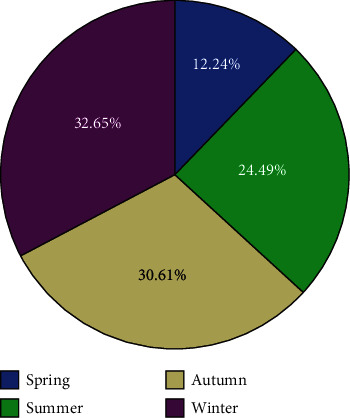
Seasonal variation of the incidence of anti-NMDAR encephalitis with ovarian teratoma.

Twenty-one patients were reviewed, of which 15 patients were admitted to the Third Affiliated Hospital, Sun Yat-sen University, and the other 6 patients were admitted to the Zhujiang Hospital, Southern Medical University, Guangzhou, China. All 21 patients were female. The mean age of patients was 24.33 ± 5.12 years (ranging from 15 to 33 years old). The disease could occur all year round, and the peak seasons focused in autumn and winter (30.61% and 32.65%) (Figure 1).

### 3.1. Clinical Features

Thirteen patients (61.9%) had prodromal symptoms, including fever and headache, and the mean duration was 7.15 ± 5.92 d, ranging from 1 d to 20 d. And 20 patients (95.2%) had psychiatric abnormalities, 14 (66.7%) patients had seizures, 12 (57.1%) patients had movement dysfunction, 11 (52.4%) patients had loss of consciousness, 11 (52.4%) patients had autonomic dysregulation, 9 (42.9%) patients had speech disturbance, 8 (38.1%) patients had central hypoventilation, and 2 (9.5%) patients had memory deficits. For all patients, 12 of them (57.1%) developed psychiatric symptoms as initial manifestations, and the other 9 cases (42.9%) presented with seizures initially. All patients developed four or more categories of symptoms within the first four weeks. Twelve of 21 patients (57.1%) had a maximum mRS of 5, and 11 of 21 patients (52.4%) were admitted to ICU.

Only one patient (4.8%) had lower abdominal pain during the onset period, as a result of acute torsion of ovarian teratoma. Other patients did not present any gynecological symptoms.

### 3.2. Ancillary Examinations

Ten of 21 patients (47.6%) had lymphocytic pleocytosis of cerebrospinal fluid. Seropositive anti-thyroid autoimmune antibodies were detected in 10 of 21 patients (47.6%). Seven of 21 patients (33.3%) had abnormalities in thyroid function test. In addition, 8 patients underwent 25-hydroxyvitamin D detection, and 7 of them (87.5%) had a lower level than normal, ranging from 18.9 to 44.0 nmol/L.

We detected serum cancer antigen 125 (CA 125), carbohydrate antigen 19-9 (CA 19-9), and serum alpha protein (AFP). And 8 of 21 patients' (38.1%) CA 125 level was higher than normal, and the maximum value was 218.6 KU/L. Three of the 21 patients (14.3%) had elevated AFP levels, and the maximum value was 34.9 ng/ml. All patients' CA19-9 values were in the normal range.

All patients received intensive oncological assessments routinely, including pelvic ultrasound and/or abdominal-pelvis CT/MRI. The diameters of the ovarian teratoma ranged from 10 to 160 mm. Nineteen of the 21 patients' (90.5%) ovarian teratomas were unilateral, and only 2 of the 21 patients' (9.5%) ovarian teratomas were bilateral.

### 3.3. Treatment and Outcomes

All patients received immunotherapy. Eight patients (38.1%) were treated only with single or multiple first-line immunotherapies, and the other 13 patients (61.9%) received first-line immunotherapies and second-line immunotherapy subsequently.

Twenty patients underwent surgery during the initial episode. Only one patient did not receive surgery because of the rejection of her family. Of the 20 patients with surgical treatment, 6 patients (30.0%) received ovarian cystectomy, and the other 14 patients (70.0%) received affected unilateral adnexectomy. The mean mRS score of patients receiving ovarian cystectomy was 3.50 ± 0.84, which was lower than that of patients receiving affected unilateral adnexectomy (4.71 ± 0.61) (*p* = 0.006). The mean duration between disease onset and surgery was 36.92 ± 14.72 d, ranging from 16 d to 63 d. Of the 20 patients who underwent surgery, 17 patients (85.0%) got clinical improvement after surgery, and the other 3 patients (15.0%) had no improvement of the mRS scores for 4 weeks after surgery.

**Figure 2:**
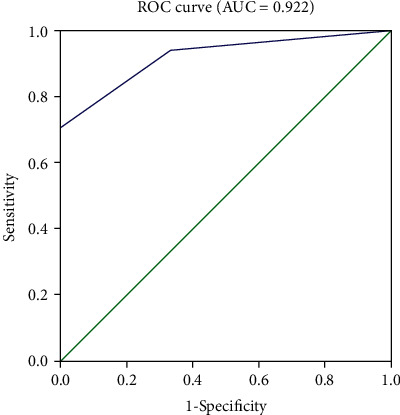
ROC curves showed the predictive efficiency of the model for the surgical outcome.

Table 1 summarized the difference of general and clinical characteristics of patients having clinical improvement and patients having no clinical improvement after surgery. There was no significant difference in age, gravidity, history of ovarian surgery, prodromal symptoms, type of initial symptoms, incidence of all other symptoms except central hypoventilation, number of symptoms, ICU admission, diameter of the ovarian teratoma, pathological type of ovarian teratoma, or duration between surgery and onset between patients having no clinical improvement after surgery and patients having clinical improvement after surgery. The incidence of central hypoventilation in patients having no clinical improvement after surgery was significantly higher than those in patients having clinical improvement after surgery (100.0% vs. 29.4%, *p* = 0.049). Additionally, the mRS score at acute onset in patients having no improvement after surgery was 5.00 ± 0.00, which was higher than that in patients having clinical improvement after surgery (4.23 ± 0.90), and the difference was statistically significant (*p* = 0.003). In multivariable analysis, the factors associated with surgical outcome included central hypoventilation (OR 0.89, 95% CI 0.60-1.45, and *p* = 0.021) and mature ovarian teratoma (OR 1.39, 95% CI 0.89-1.93, and *p* = 0.007) (Table 2). The estimated regression coefficients were as follows: logit (*p*) = −19.817^∗^*A* + 2.079^∗^*B* + 19.123, where *A* represented the symptom of central hypoventilation, and *B* represented the pathological type of mature ovarian teratoma. The ROC curve revealed that the model was reliable in predicting the surgical outcome (Figure 2).

At 12-month follow-up, all of the 17 patients having clinical improvement after surgery had a good outcome (mRS ≤ 2) and no one died or relapsed. Among 3 patients having no improvement after surgery, no one had a good outcome (mRS ≤ 2). No relapse of ovarian tumor or significant changes in menstrual pattern was observed in the 20 patients who underwent surgery.

### 3.4. Pathological Findings

**Figure 3:**
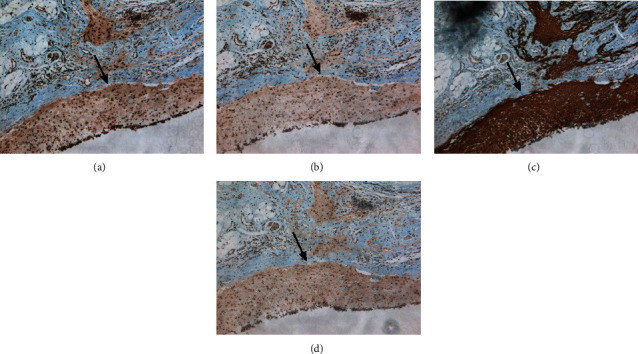
Representative immunohistochemical features. Densely aggregated neurons (the black arrow) were positive for (a) MAP2, (b) NDMAR1, (c) NDMAR2A, and (d) NDMAR2B (a–d, ×40).

**Figure 4:**
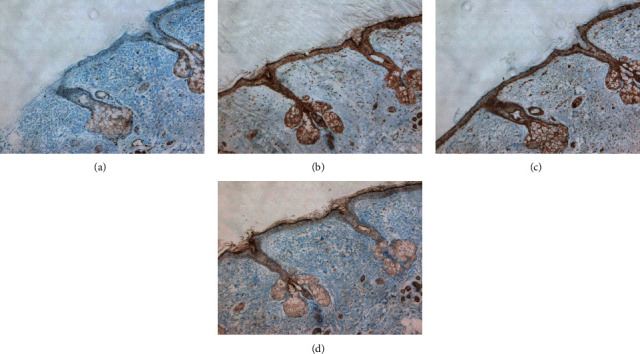
Representative immunohistochemical features. Sebaceous glands and squamous epithelial tissue were found in the tissue and were negative for (a) MAP2, strongly positive for (b) NMDAR1 and (c) NDMAR2A, and weakly positive for (d) NMDAR2B (a–d, ×40).

Seventeen of the 20 patients' (85.0%) histological types were reported to be mature ovarian teratoma, and the other 3 (15.0%) were reported to be immature ovarian teratoma. We performed immunohistochemical tests for 8 of the 20 patients' teratoma tissues. The result revealed that all 8 samples were positive for MAP2, a specific marker of mature neurons, and the same areas were also positive for NDMAR1, NMDAR2A, and NMDAR2B (Figure 3). Among which, 3 patients' ovarian teratomas also showed positive for NDMAR1, NMDAR2A, and NMDAR2B in the sebaceous glands and squamous tissues (Figure 4).

## 4. Discussion

This retrospective study demonstrates that patients with anti-NMDAR encephalitis with ovarian teratoma tend to present more neuropsychiatric symptoms than gynecological symptoms. The disease incidence is higher during autumn and winter. Most patients get clinical improvement after surgery. The central hypoventilation symptom and mature ovarian teratomas are associated with surgical outcome. Partial cases also contain large number of NMDAR-positive sebaceous glands and squamous tissues.

The mean age of the patients in this study was consistent with other studies [5]. The youngest patient in our study was 15 years old. Leel et al. reported the youngest patient with anti-NDMAR encephalitis with ovarian teratoma, who was just 7 years old [7]. So it suggests that there is a need to conduct ovarian tumors screening for female patients of all ages.

Several autoimmune diseases are known to have well-established seasonal variation. The incidence of acute gouty attacks is highest in the spring. The onset or exacerbation of rheumatoid arthritis and Wegener's granulomatosis are all seen more commonly in the winter [8]. However, there were few studies focused on the onset season of anti-NDMAR encephalitis. Adang et al. [9] revealed that anti-NDMAR encephalitis without tumor were more common in warm months (April-September) (18/23, 78%), whereas anti-NDMAR encephalitis with tumors presented during cold months (6/6,100%). Our study revealed that the onset of anti-NMDAR encephalitis with ovarian teratoma was more commonly in autumn and winter, which was the same with the current study. As the samples of both studies are limited, we still need a larger-scale study to determine whether anti-NMDAR encephalitis has seasonal variation or not.

Our data revealed that most patients presented psychiatric abnormalities as initial symptom, which was consistent with previous studies [10–12]. As to the gynecological symptoms, only one patient had lower abdominal pain. It revealed that it is important to screen tumors with imaging methods for all anti-NMDAR encephalitis patients. Ultrasound is the first choice to screen ovarian teratoma, and transvaginal ultrasound is more sensitive than transabdomial ultrasound [13]. CT or MRI has a higher sensitivity in detecting teratomas, compared to that of ultrasound [14]. However, ultrasound has many advantages, like the lower cost, shorter examination time, and convenience for the patients with serious conditions. In some cases of anti-NMDAR encephalitis, whose screening revealed no specific clue for ovarian teratoma, an occult teratoma was found by histological examination after laparoscopy examination and ovariectomy [15]. However, we could only conduct laparoscopic exploration for patients with detection of ovarian teratoma by imaging examination, because of ethical considerations.

The seropositive rate of anti-thyroid autoimmune antibodies in our study was 47.6%, which was higher than that of limbic encephalitis patients (33%) and the general population(10-15%) [16, 17]. Study found that anti-NMDAR encephalitis patients with seropositive anti-thyroid antibodies had a higher mRS score [18], but the relevant mechanism remains to be studied.

The role of B cells in the autoimmunity of anti-NMDAR encephalitis has become increasingly prominent [19–21]. Recently, the effect of vitamin D on immune cells has gradually gained attention [22, 23]. Vitamin D suppresses the proliferation and differentiation of B cells, resulting in a reduction of immunoglobulin secretion [24]. Our data found that the levels of 25-hydroxyvitamin D of most patients with anti-NMDAR encephalitis with teratoma were significantly lower than normal. It suggests that 25-hydroxyvitamin D may be an index to reflect the disease severity, which needs further study to confirm.

Early treatment and a lack of ICU admission are reported predictors of good outcome [4]. In our studies, the proportion of ICU admission was higher for patients having clinical improvement after surgery than that of the patients having improvement after surgery, but the difference had no statistical significance. Our findings suggested that the central hypoventilation symptom and mature ovarian teratomas were associated with surgical outcome of patients with anti-NMDAR encephalitis and ovarian teratoma.

Teratoma may contain tissues from all three germ layers, including ectoderm, mesoderm, and endoderm, and it can be either mature or immature. Bost et al. [25] reported that 11.8% of ovarian teratoma was immature in patients with anti-NMDAR encephalitis, which was associated with a higher risk of death. Patients with immature ovarian teratoma accounted for 10.0% in our study, but they did not present a higher mRS score or worse prognosis. In our study, all teratomas contained neural tissue, and immunohistochemical analysis confirmed that NMDAR expressed in the neural tissues, besides that NMDAR also expressed in the sebaceous glands and squamous tissue. As some patients' teratomas lack neural tissue and the majority of the teratomas are composed of skin, we speculate that encephalitis development in some patients may result from NMDAR expression in sebaceous glands and squamous epithelial tissues.

There were a few limitations in this study. First, this was a retrospective study and bias was inevitable. Second, our data only focused on the pathological results of ovarian teratoma in patients with anti-NMDAR encephalitis. Next, we plan to explore and compare the pathological features of teratomas in patients without anti-NDMAR encephalitis.

## 5. Conclusion

In summary, this study confirms that the disease incidence is higher during autumn and winter. Most patients get clinical improvement after surgery. The central hypoventilation symptom and mature ovarian teratomas are associated with surgical outcome. Systemic complications, such as pulmonary infection and respiration failure, should not be looked as surgical contraindications. We hope that our study can attract gynecologists and anesthesiologists' attention to the importance and necessity of early resection of tumors.

## Figures and Tables

**Table 1:** General and clinical characteristics in patients having clinical improvement after surgery and patients having no improvement after surgery (*N* = 20).

Parameters	Patients having improvement after surgery (*n* = 17)^§^	Patients having no improvement after surgery (*n* = 3)^€^	*t*	*p*
Age (y)	23.53 ± 5.03	28.00 ± 5.56	-1.403	0.178
Fertilized women	3 (17.6%)	2 (66.7%)		0.140^∗^
History of ovarian surgery	2 (11.8%)	0 (0.0%)		0.716^∗^
Clinical features [5]				
Prodromal symptoms				
Fever (*T* > 37.5°C)	4 (23.5%)	1 (33.3%)		0.601^∗^
Headache	6 (35.3%)	2 (66.7%)		0.620^∗^
Initial symptoms				0.579^∗^
Psychiatric abnormalities	9 (52.9%)	2 (66.7%)		
Seizures	8 (47.1%)	1 (33.3%)		
Psychiatric abnormalities	17 (100.0%)	3 (100.0%)		-
Seizures	12 (70.6%)	2 (66.7%)		0.681^∗^
Movement dysfunction	10 (58.8%)	2 (66.7%)		0.656^∗^
Loss of consciousness	8 (47.1%)	3 (100.0%)		0.218^∗^
Autonomic dysregulation	10 (58.8%)	1 (33.3%)		0.566^∗^
Speech disturbance	6 (35.3%)	2 (66.7%)		0.537^∗^
Central hypoventilation	5 (29.4%)	3 (100.0%)		0.049^∗^
Memory deficits	2 (11.8%)	0 (0.0%)		0.716^∗^
Number of symptoms^#^ [4]	3.94 ± 1.39	5.33 ± 1.15	-1.627	0.121
ICU admission [4]	8 (47.1%)	3 (100.0%)		0.218^∗^
mRS at onset [4]	4.23 ± 0.90	5.00 ± 0.00	-3.490	0.003
Diameter of the ovarian teratoma (mm)	48.47 ± 35.31	49.00 ± 13.53	-0.025	0.980^∗^
Pathological type				0.284^∗^
Mature ovarian teratoma	16 (94.1%)	2 (66.7%)		
Immature ovarian teratoma	1 (5.9%)	1 (33.3%)		
Time until surgery initiation (d)	36.10 ± 13.22	45.67 ± 21.22	-0.969	0.353^∗^

^§^A decrease in mRS ≥ 1 was defined as having clinical improvement after surgery. ^**€**^No change in mRS or mRS ≥ 4 for 1 month after surgery was defined as having no clinical improvement. ^#^The eight symptom categories are as follows: psychiatric abnormalities, seizures, movement dysfunction, loss of consciousness, autonomic dysregulation, speech disturbance, central hypoventilation, and memory deficits. ^∗^Fisher's exact test. mRS: modified Rankin Scale; ICU: intensive care unit.

**Table 2:** Factors associated with clinical improvement after surgery (multivariable analysis).

Factor	*p*	OR	95% CI
Age	0.160		
Fertilized women	0.718		
Loss of consciousness	0.089		
Central hypoventilation	0.021	0.89	0.60-1.45
Number of symptoms	0.109		
ICU admission	0.089		
mRS at onset	0.152		
Pathological type			
Mature ovarian teratoma	0.007	1.39	0.89-1.93

OR: odds ratio; CI: confidence interval; mRS: modified Rankin Scale; ICU: intensive care unit.

## Data Availability

The data used to support the findings of this study are included within the article.
